# Privacy, Proficiency, and the Promise of Wearables: Insights from Focus Groups with Older Adults

**DOI:** 10.1093/geroni/igaf122.2812

**Published:** 2025-12-31

**Authors:** Justin Lam, Walter Boot

**Affiliations:** Donald and Barbara Zucker School of Medicine at Hofstra/Northwell, Brooklyn, New York, United States; Weill Cornell Medicine, New York, New York, United States

## Abstract

Older adults are increasingly using the Internet, laptops, smartphones, and other digital devices to access healthcare services and information. Additionally, wearable technologies offer the potential to continuously monitor health data, which could be used to detect and address age-related health issues. However, the adoption of these technologies remains challenging due to difficulties in using the technology and concerns about privacy. In this study, we build upon the results of Fowe and Boot’s (2022) survey study, which examined older adults’ attitudes toward the use of wearable and mobile technologies for predicting cognitive decline, supporting healthy behaviors, and collecting self-reported health data. The current study aimed to qualitatively explore the barriers and facilitators of wearable technology use. Data were collected through four focus group sessions with older adults. Thematic analysis revealed five main themes: (1) privacy concerns, (2) digital “nagging,” (3) accuracy and reliability concerns, (4) proficiency challenges, and (5) a strong interest in monitoring personal health. These findings underscore the importance of developing resources that enhance older adults’ skills, confidence, and self-efficacy in using wearable technologies safely and effectively.

